# Metagenomic Profiling of Fecal and Cecal Microbiota and Their Antimicrobial Resistance Genes in Indigenous Backyard Poultry

**DOI:** 10.1155/ijm/7306065

**Published:** 2026-01-25

**Authors:** Philip M. Panyako, Stephen Ogada, Stephen N. Kuria, John Musina, Jacqueline K. Lichoti, Sheila C. Ommeh

**Affiliations:** ^1^ Institute for Biotechnology Research, Jomo Kenyatta University of Agriculture and Technology, Nairobi, Kenya, jkuat.ac.ke; ^2^ The University of Queensland, Queensland Alliance for Agriculture & Food Innovation, Centre for Animal Science, St Lucia, Queensland, Australia, uq.edu.au; ^3^ Department of Zoology, National Museums of Kenya, Nairobi, Kenya, museums.or.ke; ^4^ State Department of Livestock Development, Ministry of Agriculture and Livestock Development, Nairobi, Kenya

**Keywords:** AMR, antibiotics, antimicrobial stewardship, diagnostic stewardship, gut microbiota, public health, shotgun metagenomics

## Abstract

Indigenous backyard poultry is the predominant type of poultry in developing countries. Rural smallholder farmers in these regions usually adopt the free‐range (backyard) production system, which exposes the poultry to diverse environments and a broad spectrum of microorganisms that influence their diet and gut microbiota. In this cross‐sectional purposive study, we evaluated the microbial community profiles of indigenous backyard poultry and their antimicrobial resistance genes (ARGs) using both cecal samples, which provide a more accurate representation of the core gut microbiota, and fecal samples, which allow for noninvasive monitoring and pathogen screening. We analyzed 32 pooled fecal and cecal samples using shotgun metagenomics, followed by functional and antimicrobial resistance (AMR) analyses to identify genes and metabolic pathways associated with poultry gut health and production. We report the presence of many commensal microorganisms in indigenous backyard poultry, with the most abundant being Bacteroidetes, Firmicutes, and Proteobacteria. The most dominant genera in the feces were *Bacteroides*, *Methanobrevibacter*, and *Phocaeicola*, while *Bacteroides*, *Methanobrevibacter*, and *Chlamydia* dominated in the ceca. No marked differences in microbial diversity were observed between the fecal and cecal samples. KEGG and COG database analyses revealed significantly enriched pathways associated with metabolism, cellular processes, and information storage and processing. Genes that confer resistance to tetracycline were the most abundant, raising concerns about the risks associated with inappropriate and excessive use of this antibiotic in poultry treatment. These findings deepen our understanding of the poultry gut microbiome, particularly regarding indigenous backyard poultry. Furthermore, the information about ARGs is a valuable indicator of antimicrobial use by rural smallholder farmers who have adopted the free‐range production system in Kenya and other developing countries. These insights are crucial for farmers and the national livestock sector to monitor AMR in poultry, thereby enabling improved poultry management practices and informed policy decisions.

## 1. Introduction

Poultry farming is essential for the livelihoods of many rural smallholder farmers in Africa and other developing regions worldwide, as it enhances human nutrition, generates income, and provides manure for crop production [1]. The poultry population in Kenya is estimated to exceed 50 million birds, with indigenous chickens forming the largest proportion (75%), followed by commercial layers and broilers at 24% and other poultry species at 2% [2, 3]. In Kenya and other developing countries, indigenous poultry is mainly raised under the free‐range (backyard or scavenging) production system in rural smallholder farm settings, thus commonly called village or indigenous backyard poultry [4]. The free‐range production system is popular in rural areas because it is less capital‐intensive and requires little to no biosecurity and biosafety measures, which can be costly [5]. The birds are typically allowed to roam freely, feeding on vegetation, seeds, fruits, soil particles, arthropods, and worms, which reduces their feeding costs [6, 7]. Indigenous backyard poultry are thus exposed to a wide variety of microorganisms because of their diverse diet, which affects the composition and diversity of their gut microbiota.

The poultry gut microbiota is a community of microorganisms, including bacteria, archaea, fungi, algae, protozoa, and viruses. They are typically introduced into their hosts through the gastrointestinal tract (GIT), where they multiply [8]. The interaction between these microorganisms, along with their collective genetic material, plays a crucial role in the overall health and function of the host [9]. Microorganisms have been shown to contribute to the health of the host by establishing either a symbiotic, commensal, or pathogenic relationship [10]. Due to the pathogenic potential of some poultry microorganisms, poor rural smallholder farmers often administer antibiotics indiscriminately to poultry that show any signs of illness [11]. Unfortunately, this is usually done without determining whether the etiological agent involved is viral or bacterial. This improper and unregulated use of antibiotics for treating poultry diseases has resulted in antimicrobial resistance (AMR) among many poultry in these rural areas. Therefore, it is essential to assess the gut microbial community profiles of indigenous backyard poultry along with their exposure to AMR.

Several approaches have been used to study the gut microbiota of poultry. Unfortunately, while culture‐based methods are instrumental and useful, they can be biased and inaccurate in some instances because many microorganisms cannot be cultivated, and their growth requirements remain unknown [9]. Metagenomics‐based detection methods are currently being used successfully to characterize microbial communities in various hosts and environments [12]. The popularity of these techniques has largely stemmed from their high sensitivity and broad coverage, targeting the entire genome [13]. Therefore, they can be used to detect all microorganisms present in the sample of interest. Additionally, less abundant taxa and their functional potential can be identified, which helps reveal more microbial diversity within and between samples [14].

Most metagenomic studies of poultry microbiomes have been conducted on poultry raised under controlled and regulated feeding regimes [15–19]. However, few studies have examined the microbial community profiles of indigenous backyard poultry raised in a free‐range production system [20]. Feces were specifically chosen for shotgun metagenomics analysis because they are noninvasive and easy to collect, allowing for continuous observation of changes over time without the need for complicated sample collection procedures, such as sacrificing the birds [21]. In contrast, the digestion of oligosaccharides and other complex polysaccharides occurs in the cecum and is closely linked to cecal microbiota [21]. Therefore, studying the fecal and cecal gut microbiomes in indigenous backyard poultry and their interactions with the host can help develop new dietary or management interventions that can enhance their growth, optimize feed utilization, and provide insights into the proper use of antimicrobial agents.

## 2. Materials and Methods

### 2.1. Sample Collection and Pooling

**Figure 1:**
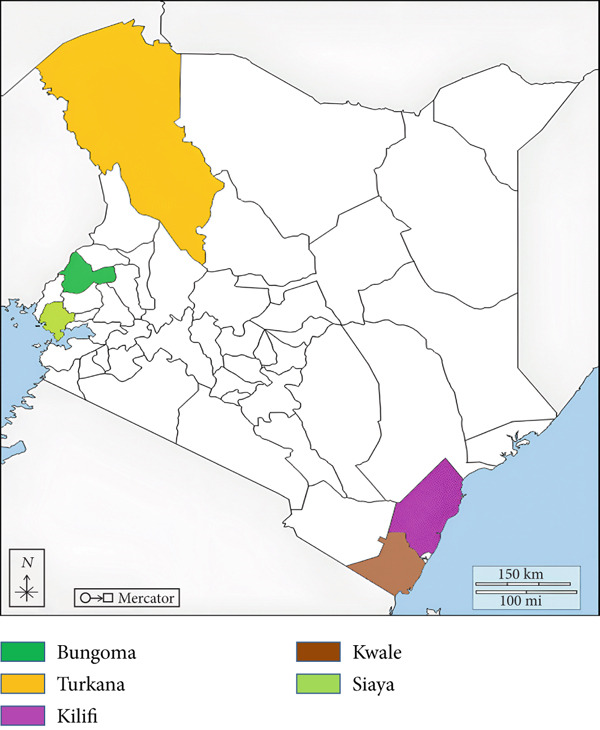
Map of Kenya showing the main sampling sites with high concentrations of indigenous backyard poultry and diverse agroclimatic conditions. *Source:* https://d-maps.com/.

Sampling was conducted from October to December 2018 across six counties in Kenya with varying agroecological conditions (Figure 1). The targeted regions included counties bordering Uganda (Siaya, Bungoma, and Turkana) and maritime borders (Kilifi and Kwale). Farmers in these regions adopt various poultry production systems that are significantly influenced by resource availability as well as geographic and climatic conditions. The study received institutional approval from Jomo Kenyatta University of Agriculture and Technology (JKUAT) for animal research. Approval to study farm animals was also obtained from the Directorate of Veterinary Services of the State Department for Livestock Development, Ministry of Agriculture and Livestock Development, Kenya.

A cross‐sectional, stratified, and purposive sampling approach was adopted during the study. Fresh fecal samples (*n* = 48) were collected from the indigenous backyard poultry, which had rested overnight before they were humanely euthanized using killing cones for cecal sample collection (*n* = 48). The samples, which were collected in 2‐mL Eppendorf tubes, were immediately frozen in dry ice and then placed in liquid nitrogen and transported to JKUAT, Kenya, for storage at −80°C and laboratory work.

Out of the 48 samples collected for each sample type, three were pooled together (based on the poultry species and sex) to create a single pooled sample. This method was applied to fecal and cecal sample types, resulting in 32 pools: 16 fecal and 16 cecal pools (Table 1). Each pool was created by thoroughly mixing contents from the corresponding individual samples to ensure homogeneity. This pooling strategy was designed to minimize variability of the samples for successive metagenomic analyses.

**Table 1:** Pooled cecal and fecal samples collected from indigenous backyard poultry (chickens, ducks, pigeons, and guinea fowls).

**Pooled sample ID**	**Sample type**	**Species**	**Sex**	**Region**
S001	Fecal	Chicken	Female	Siaya
S002	Cecal	Chicken	Female	Siaya
S003	Fecal	Chicken	Male	Siaya
S004	Cecal	Chicken	Male	Siaya
S005	Fecal	Chicken	Female	Laboot
S006	Cecal	Chicken	Female	Laboot
S007	Fecal	Chicken	Male	Laboot
S008	Cecal	Chicken	Male	Laboot
S009	Fecal	Chicken	Female	Turkana
S010	Cecal	Chicken	Female	Turkana
S011	Fecal	Chicken	Male	Turkana
S012	Cecal	Chicken	Male	Turkana
S013	Fecal	Chicken	Female	Kilifi
S014	Cecal	Chicken	Female	Kilifi
S015	Fecal	Chicken	Male	Kilifi
S016	Cecal	Chicken	Male	Kilifi
S017	Fecal	Chicken	Female	Kwale
S018	Cecal	Chicken	Female	Kwale
S019	Fecal	Chicken	Male	Kwale
S020	Cecal	Chicken	Male	Kwale
S021	Fecal	Duck	Female	Bungoma
S022	Cecal	Duck	Female	Bungoma
S023	Fecal	Duck	Male	Bungoma
S024	Cecal	Duck	Male	Bungoma
S025	Fecal	Pigeon	Female	Bungoma
S026	Cecal	Pigeon	Female	Bungoma
S027	Fecal	Pigeon	Male	Bungoma
S028	Cecal	Pigeon	Male	Bungoma
S029	Fecal	Guinea fowl	Female	Bungoma
S030	Cecal	Guinea fowl	Female	Bungoma
S031	Fecal	Guinea fowl	Male	Bungoma
S032	Cecal	Guinea fowl	Male	Bungoma

### 2.2. DNA Extraction

DNA was extracted from the pooled fecal and cecal samples using the PureLink Genomic DNA Mini Kit (Invitrogen, Thermo Fisher Scientific, Waltham, Massachusetts, United States) according to the manufacturer′s protocol as described in Panyako [22]. Briefly, 200 *μ*L of phosphate‐buffered saline (PBS) and 20 *μ*L of proteinase K were added to the pooled samples and mixed well by pipetting. An equal volume (200 *μ*L) of PureLink Genomic Lysis/Binding Buffer was added to the lysate and mixed well by vortexing briefly before incubating at 55°C for at least 10 min. The lysate was briefly centrifuged at 3000 × *g*, and 200 *μ*L of 99% ethanol was added and mixed well by vortexing for 5 s. The lysate was then added to a PureLink Spin Column attached to a collection tube and centrifuged at 10,000 × *g* for 1 min at room temperature. The collection tube was discarded, and the spin column was placed into a clean PureLink collection tube. To wash the extracted DNA, 500 *μ*L of the Wash Buffer 1, prepared with ethanol, was added to the column and centrifuged at room temperature at 10,000 × *g* for 1 mine. The collection tube was discarded, and the spin column was placed into a clean PureLink collection tube. A second wash was performed by adding 500 *μ*L of Wash Buffer 2 to the column and centrifuging at maximum speed for 3 min at room temperature. The collection tube was then discarded. The spin column was finally placed in a sterile 1.5‐mL microcentrifuge tube, and 50 *μ*L of PureLink Genomic Elution Buffer was added to the column, which was incubated at room temperature for 1 min and centrifuged at maximum speed for 1 min at room temperature. To recover more DNA, a second elution step using the same elution buffer volume as the first was performed in another sterile, 1.5‐mL microcentrifuge tube. The column was then removed and discarded. The purified DNA solution was stored in a −20°C freezer until processing.

### 2.3. Library Preparation and Sequencing

The quality and quantity of the DNA preparations were determined in the NanoDrop 2000 spectrophotometer and Qubit fluorometer (Invitrogen, Thermo Fisher Scientific Inc., Waltham, Massachusetts, United States), respectively. The extracted genomic DNA was used to prepare indexed paired‐end libraries using the Nextera XT DNA Library Preparation Kit according to the manufacturer′s instructions (Illumina Inc., United States). Indexed samples were pooled and reconstituted to 4 nM before diluting to 12 pM for loading into the MiSeq instrument (Illumina, California, United States) Version 2 reagent kit (300 cycles) with a paired‐end format (2 × 150 cycles) at the ILRI Genomic platform, Nairobi, Kenya.

### 2.4. Bioinformatics and Statistical Analyses

The paired‐end raw sequence reads were subjected to quality checks using FastQC Version 0.11.9 [23] to ensure only high‐quality reads were retained for subsequent analyses. Reads shorter than 50 base‐pairs, poor‐quality reads with a Phred quality score of < 20, and adapter sequences were trimmed out using PRINSEQ Version 0.39 [24]. The filtered sequence reads were mapped against host reference sequences to remove poultry host DNA sequence data using Bowtie2 Version 2.4.5 [25], followed by additional quality checks using FastQC. To improve metagenomic taxonomic profiling and functional annotation, reference‐ and assembly‐based approaches were used as described by Kiige [26].

MetaPhlAn Version 3.0 [27], which maps individual sequence reads against a clade‐specific marker gene reference database, was used to generate the relative abundances of the microbiota present in each sample. The relative abundance reports of the samples were merged and analyzed in R programming software. Rarefaction analysis was done using the *vegan* R package to control for variation in uneven sequencing effort when measuring alpha and beta diversity indices. The relative abundances of bacteria and archaea at each taxonomic level were visualized using bar plots and cluster maps generated with the *ggplot2* and *pheatmap* R packages in R Version 4.3.0 [28]. Alpha diversity indices (Chao1, ACE, Shannon, Simpson, and Inverse Simpson), which measure species richness, evenness, and overall diversity within samples, were calculated and visualized using the R packages *phyloseq* and *ggplot2*. Statistical analysis of alpha diversity was performed using the nonparametric Wilcoxon rank‐sum (Mann–Whitney) test to compare diversity between the sample types. Bray–Curtis and Jaccard dissimilarity metrics (distances) were used for beta diversity analysis to measure the distance between the bacterial compositions of fecal and cecal sample types. The distances between samples were then visualized using principal coordinate analysis (PCoA) and nonmetric multidimensional scaling (NMDS). The statistical significance of the beta diversity analyses was estimated using a permutational multivariate analysis of variance (PERMANOVA) with 999 permutations using the *phyloseq* R package.

The SqueezeMeta pipeline, which follows an assembly‐based approach, was run in the *coassembly* mode. Here, the sequence reads from all samples are pooled, and a single assembly is performed, followed by mapping of the reads to the *coassembly* to obtain gene abundances. Assembly of the sequence reads and taxonomic assignment were performed using MEGAHIT [29] and RDP Classifier [30], respectively. The gene‐finding algorithm Prodigal [31] was used to identify protein‐coding genes in the metagenome data. The functions of the predicted genes were inferred by comparing them against taxonomic and functional databases, including the nonredundant (nr), Clusters of Orthologous Genes (COG), and the Kyoto Encyclopedia of Genes and Genomes (KEGG) [32], using Diamond [33] with a cutoff above 40% for the reference and query ratio.

The antimicrobial resistance genes (ARGs) from the poultry fecal and cecal content were characterized to explore the relationship between diverse sequences and resistance levels. The assembled contigs of fecal and cecal samples of the different poultry species were aligned against the NCBI AMRFinderPlus [34] and Resfinder [35] databases for mass screening of the assembled contigs for ARGs using ABRicate Software Version 1.0.1 [36]. Based on raw read counts, the relative abundances of AMR genes were estimated. The analysis and visualization of the results in graphs and heatmaps were performed using the *vegan* and *ggplot2* R packages.

## 3. Results

### 3.1. Overview of the Sequence Data

The sequencing of cecal and fecal samples yielded 32,149,062 raw reads, with a median length of 200 base pairs (Table 2). After filtering, 30,795,227 reads were retained and assembled into 139,889 contigs. Using a 95% similarity cut‐off, the assembled contigs yielded 4437 operational taxonomic units (OTUs). Three samples (S002, S023, and S027) were not informative as they generated no OTUs that could be used for further analysis. It was not possible to obtain any significant amount of DNA from these samples, possibly due to degradation.

**Table 2:** Raw reads, filtered reads, contigs, and observed OTUs of each sample.

**Sample**	**Raw reads**	**Clean reads**	**Contigs**	**Observed OTUs**
S001	2,616,330	2,132,568	5043	143
S003	2,092,898	1,783,204	6321	138
S004	1,515,614	1,287,206	1439	158
S005	434,508	430,826	781	165
S006	101,244	96,426	89	145
S007	166,114	160,348	182	175
S008	368,138	356,246	862	165
S009	1,652,376	1,635,568	7304	132
S010	162,600	161,902	58	169
S011	993,492	987,814	3724	123
S012	3,470,728	3,456,992	20,606	176
S013	1,604,804	1,596,040	3898	173
S014	650,780	645,278	3571	169
S015	748,964	741,216	3698	157
S016	1,026,998	1,005,767	8639	146
S017	1,347,408	1,321,506	12,081	144
S018	305,850	290,024	743	148
S019	845,996	837,518	3529	159
S020	1,172,660	1,163,488	6353	155
S021	590,996	586,506	1503	148
S022	1,070,720	1,053,314	5617	133
S024	845,972	819,922	2331	135
S025	1,899,686	1,866,204	7739	141
S026	963,508	955,602	2640	166
S028	1,673,198	1,664,542	4910	189
S029	817,870	803,256	7343	145
S030	1,291,674	1,281,742	6643	150
S031	983,614	957,934	6099	149
S032	734,322	716,268	6143	141
Total	32,149,062	30,795,227	139,889	4437

**Figure 2:**
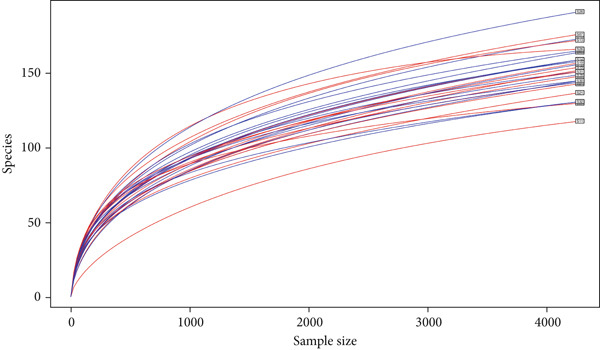
Rarefaction curves of samples clustered at 90% sequence identity. The rarefaction curves for each sample were plotted without replacement.

Rarefaction curves generated from the OTUs indicate that most samples shared similar values of observed species richness and tended to plateau, suggesting that the sample depth was adequate for estimating fecal and cecal taxa, and increasing the number of reads would have had minimal impact on species richness (Figure 2).

### 3.2. Alpha and Beta Diversity Analysis

Analysis of the alpha diversity indices (observed number of OTUs, Chao1, ACE, Shannon, and Inverse Simpson) revealed no major differences in microbiota richness, diversity, and evenness within the fecal and cecal sample types (Table 3). This implies that the species richness, diversity, and evenness of the microbiota in indigenous backyard poultry feces and those found in the cecum are generally similar.

**Table 3:** Alpha diversity indices of cecal and fecal samples.

**Sample**	**Species**	**Sample type**	**Observed OTUs**	**Chao1**	**ACE**	**Shannon**	**Inverse Simpson**
S001	Chicken	Fecal	143	195.56	196.91	3.443	16.26
S003	Chicken	Fecal	138	177.00	180.45	3.232	12.94
S004	Chicken	Cecal	158	245.00	237.52	3.085	9.202
S005	Chicken	Fecal	165	220.00	233.47	3.368	14.31
S006	Chicken	Cecal	145	179.17	185.12	3.456	17.76
S007	Chicken	Fecal	175	228.00	237.30	3.709	22.66
S008	Chicken	Cecal	165	210.72	228.87	3.663	23.65
S009	Chicken	Fecal	132	215.15	188.95	2.785	6.912
S010	Chicken	Cecal	169	242.20	252.33	3.438	16.49
S011	Chicken	Fecal	123	168.12	177.96	1.867	2.874
S012	Chicken	Cecal	176	244.44	239.06	3.500	18.24
S013	Chicken	Fecal	173	252.57	246.63	3.529	19.14
S014	Chicken	Cecal	169	228.40	230.26	3.429	16.10
S015	Chicken	Fecal	157	202.04	207.36	3.609	20.11
S016	Chicken	Cecal	146	214.06	212.35	3.592	19.81
S017	Chicken	Fecal	144	198.67	191.68	3.599	18.86
S018	Chicken	Cecal	148	178.10	194.32	3.206	12.07
S019	Chicken	Fecal	159	244.55	239.81	3.380	14.57
S020	Chicken	Cecal	155	185.88	187.80	3.483	15.67
S021	Duck	Fecal	148	229.05	225.25	3.284	12.06
S022	Duck	Cecal	133	149.61	164.46	3.381	16.75
S024	Duck	Cecal	135	192.40	180.11	3.381	16.05
S025	Pigeon	Fecal	141	171.57	175.65	2.805	6.099
S026	Pigeon	Cecal	166	168.63	173.50	3.444	10.45
S028	Pigeon	Cecal	189	262.20	260.18	3.586	14.54
S029	Guinea fowl	Fecal	145	202.50	207.51	3.711	24.83
S030	Guinea fowl	Cecal	150	189.05	195.01	3.532	17.27
S031	Guinea fowl	Fecal	149	198.14	219.13	3.681	21.51
S032	Guinea fowl	Cecal	141	192.48	197.55	3.340	14.80

The distribution of alpha diversity indices was also visualized with boxplots (Figure 3). The differences in median alpha diversity values were generally small across the sample types. However, fecal samples consistently show higher alpha diversity, richer and more even microbial communities, across multiple indices.

Figure 3The distribution of alpha diversity indices across (a) sample types and (b) indigenous backyard poultry species.

**Figure:**
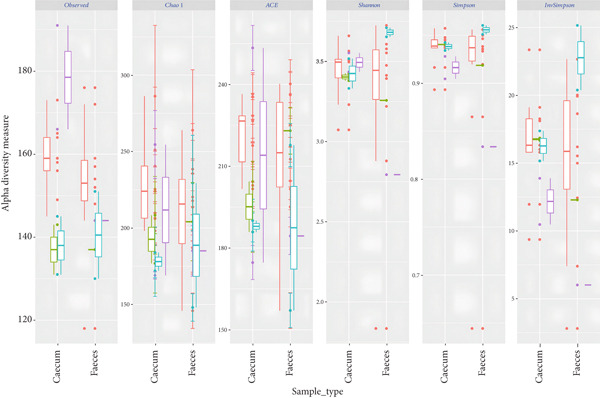
(a)

**Figure:**
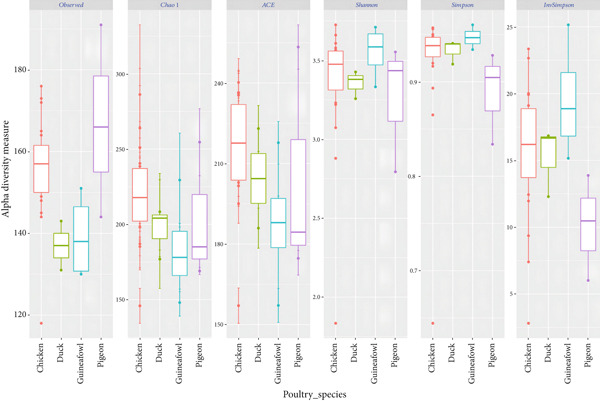
(b)

**Figure 4:**
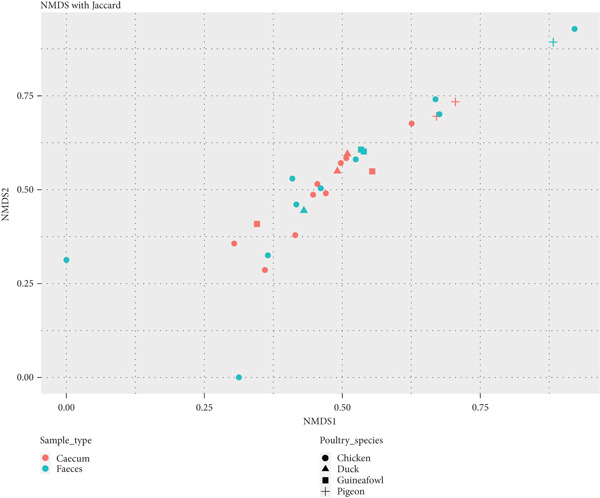
Diversity of microbiota between fecal and cecal samples across indigenous backyard poultry species, shown on the NMDS plot with Jaccard distance.

Statistical analysis of alpha diversity was conducted to examine whether there were significant differences in species richness and diversity among the sample types based on the observed number of OTUs, Shannon diversity, and Chao1 indices (Tables S1, S2, and S3, respectively). Pairwise comparisons using the Wilcoxon rank‐sum test with continuity correction revealed nonsignificant differences in metagenome species richness between fecal and cecal sample types (Holm‐adjusted, *p* > 0.05). For beta diversity analysis, the NMDS plot, used for dimension reduction analysis, showed no clear distinction between the bacterial compositions of the fecal and cecal samples (Figure 4).

**Figure 5:**
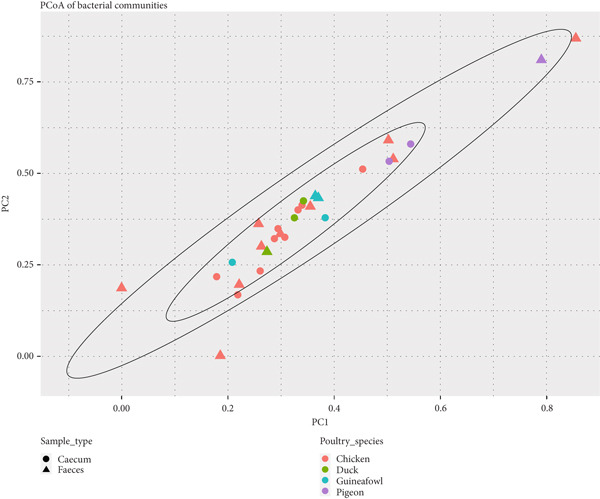
Diversity of microbiota between fecal and cecal samples across indigenous backyard poultry species, shown on the PCoA plot with Bray–Curtis distance.

No clear difference was also observed between the microbiota in the feces and ceca along Principal Coordinate Axis 1 (PC1) of the PCoA plot. The sample types primarily clustered together, suggesting that the bacterial composition among the samples was largely similar (Figure 5).

The separation was confirmed using the permutational analysis of variance (PERMANOVA), which tests whether the sample types differ significantly. PERMANOVA using the Bray–Curtis dissimilarity index revealed no significant differences in microbial composition between the cecal and fecal samples (Table 4). This finding was further supported by the PERMANOVA conducted with the Jaccard dissimilarity index (Table 5).

**Table 4:** Permutational analysis of variance testing using Bray–Curtis dissimilarity index.

	**Df**	**SumsOfSqs**	**MeanSqs**	**F.model**	**R** ^2^	**Pr (**>**F** **)**
Sample type	1	0.07621	0.076208	0.68156	0.02462	0.709
Residuals	27	3.01898	0.111814		0.97538	
Total	28	3.09519			1.00000	

**Table 5:** Permutational analysis of variance testing using the Jaccard dissimilarity index.

	**Df**	**SumsOfSqs**	**MeanSqs**	**F.model**	**R** ^2^	**Pr (**>**F** **)**
Sample type	1	0.1268	0.12680	0.66587	0.02407	0.878
Residuals	27	5.1414	0.19042		0.97593	
Total	28	5.2682			1.00000	

### 3.3. Fecal and Cecal Microbiota Composition Across Poultry Species

The phylum, class, order, family, and genus‐level abundance and distributions for individual samples are shown in Figure 6. In total, 13 and 15 phyla were identified in the fecal and cecal samples of the various poultry species, respectively. Bacteroidetes, Firmicutes, and Proteobacteria were the most dominant phyla in both fecal and cecal samples across the poultry species, indicating no significant differences in detected phyla in the fecal and cecal samples. Chicken fecal and cecal samples exhibited the highest number of phyla compared to other poultry species. Twenty‐one classes of microbes were found in both fecal and cecal samples among the different poultry species, with Bacteroidia, Methanobacteria, and Clostridia being the most dominant in chickens, ducks, and guinea fowls. A total of 27 orders were identified across the different poultry species, with the dominant orders being Bacteroidales, Methanobacteriales, and Eubacteriales.

Figure 6Relative abundance and distribution of the dominant bacterial microbiota in cecal and fecal samples of indigenous backyard poultry. Relative abundance at the (a) phylum level, (b) family level, and (c) genus level.

**Figure:**
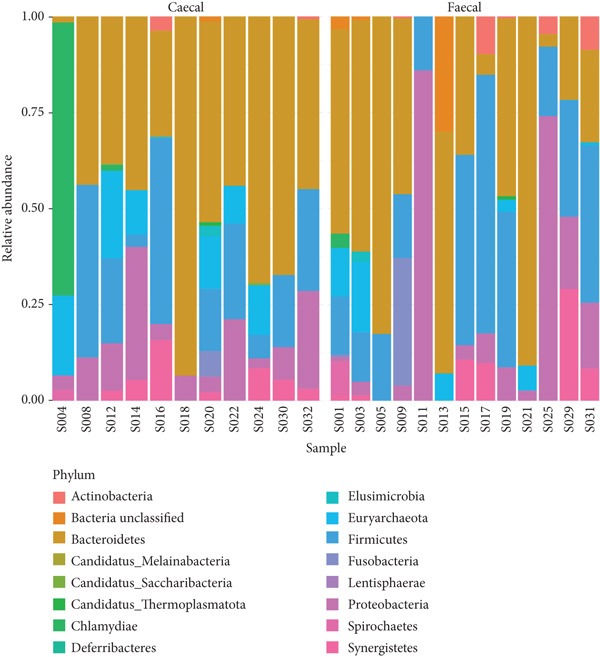
(a)

**Figure:**
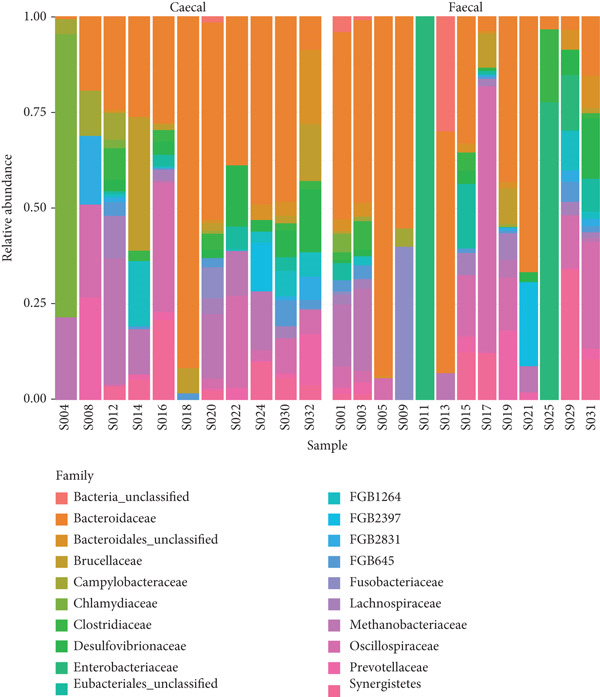
(b)

**Figure:**
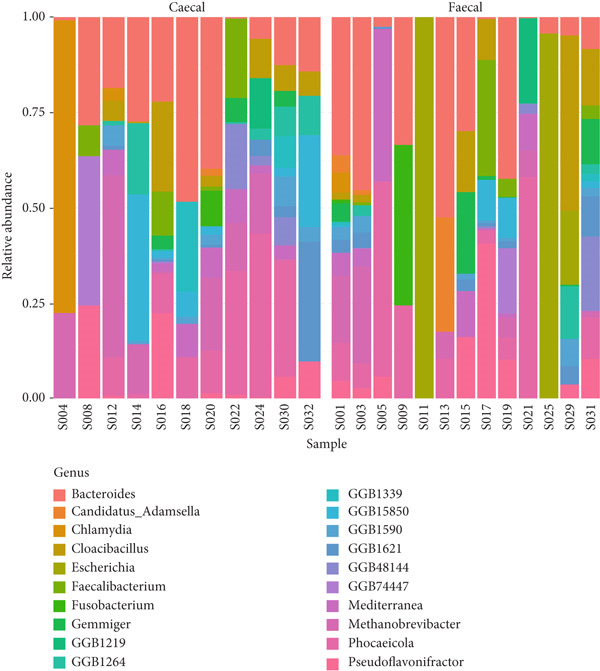
(c)

At the family level, 37 and 33 taxa were identified in fecal and cecal samples, respectively, across the poultry species. The most dominant families were Bacteroidaceae, Oscillospiraceae, and Methanobacteriaceae. A total of 61 and 57 genera were identified in fecal and cecal samples, respectively, across the poultry species. There was no significant difference in the distribution of genera detected in fecal and cecal samples. However, *Chlamydia*, *Faecalibacterium*, and *Prevotella* were more enriched in the cecal samples.

A total of 95 and 89 species were identified in both fecal and cecal samples, respectively, across the different poultry species. The most dominant bacterial species in fecal and cecal samples were *Bacteroides caecigallinarum* and *Methanobrevibacter woesei*. Hence, there was no marked difference in microbial abundances between fecal and cecal samples of the studied poultry.

**Figure 7:**
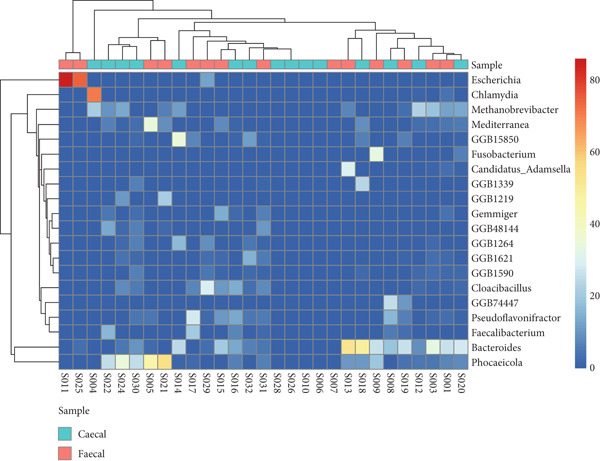
Heatmap of the relative abundance of the dominant bacterial genera in feces and ceca of indigenous backyard poultry. The color scale represents abundance from high (red) to low (blue).

To assess the relatedness and overall taxonomic similarities between the identified sequences in the fecal and cecal samples, a hierarchical clustering analysis of the dominant genera and species of all samples for both groups was performed (Figure 7). The hierarchical cluster maps for both groups generally displayed dendrograms with intermingled branches, indicating a lack of clear separation between sample types and the different indigenous backyard poultry species. The results, therefore, indicate a lack of bacterial host specificity for most of the samples studied. However, certain microorganisms were detected only in feces and not in the ceca and vice versa. The hierarchical cluster maps also revealed the dominance of *Bacteroides*, *Methanobrevibacter*, *Phocaeicola*, *Candidatus adamsella*, *Mediterranea*, and *Pseudoflavonifractor* in feces. In contrast, the most dominant genera in the ceca were *Bacteroides*, *Methanobrevibacter*, *Chlamydia*, *Pseudoflavonifractor*, *Elusimicrobium*, *Candidatus Alloclostridium*, *Faecalibacterium*, and *Prevotella*. Species abundance was also resolved, revealing that *Bacteroides caecigallinarum*, *Methanobrevibacter woesei, Phocaeicola barnesiae*, and *Bacteroides* sp. *An322* were the dominant bacterial species in feces, while *Methanobrevibacter woesei* and *Bacteroides caecigallinarum* dominated the ceca.

### 3.4. Functional Annotation

We mapped protein‐coding sequences to the KEGG and COG databases, and the relative abundance of Level 1 hits in each database was represented as a heatmap of functional abundance for each sample (Figure 8).

Figure 8Heatmap showing the relative abundance of functional genes present in the ceca and feces of indigenous backyard poultry based on the (a) KEGG and (b) COG databases. The color scale represents the relative abundance, ranging from navy blue (high abundance) to lavender (low abundance).

**Figure:**
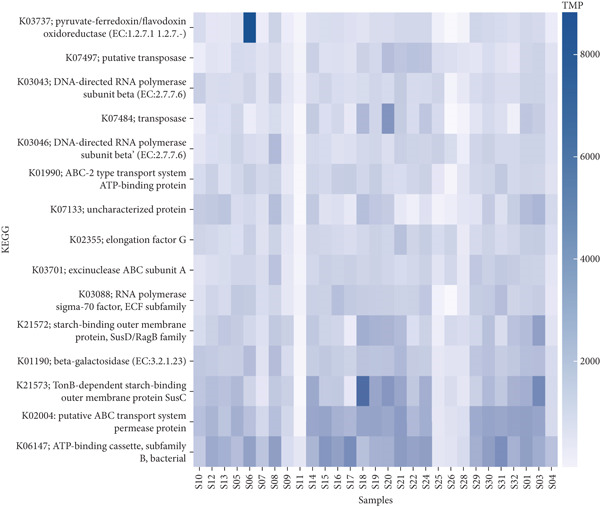
(a)

**Figure:**
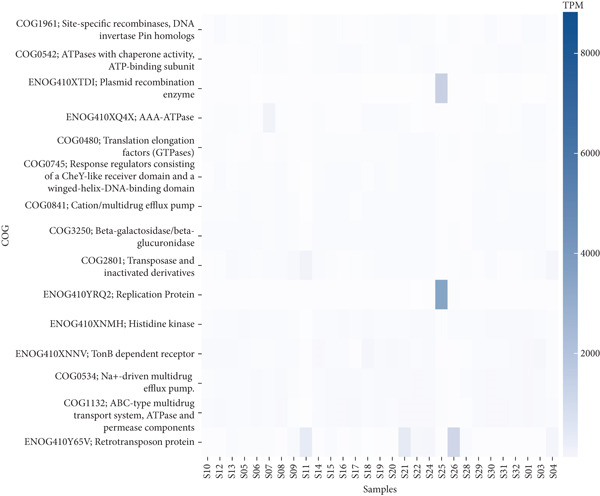
(b)

The KEGG pathway analysis indicated that functions such as transposition, metabolism, cellular processes, and human diseases were predicted in the fecal and cecal samples. Conversely, the COG pathway analysis showed that cellular processes and signaling, information storage and processing, and metabolism were detected in the fecal and cecal samples.

### 3.5. Detection of ARGs in Feces and Ceca

The Antimicrobial Resistance Genes Database (ARDB) was used to identify ARGs in the fecal and cecal samples from all poultry species. The primary ARGs detected in the fecal samples are shown in Figure 9. The bar graph and heatmap reveal a variable abundance of ARGs across the samples. Most of the identified ARGs conferred resistance to tetracycline (*tetW3*, *tetQ1*, *tetA*, *tetW1*, and *tetW5*). The *tetQ1* and *tetW1* ARGs were present in most poultry fecal samples, indicating that these genes are the most common tetracycline‐resistant ones. Other ARGs identified in some samples include those conferring resistance to *β*‐lactamases (*bla_OXA851_*), aminoglycosides (*aph6ld1* and *aph3lb1*), and sulfonamides (*sul211*), although these classes of antibiotics were only found in chicken fecal samples.

Figure 9Total level of antimicrobial resistance genes in fecal samples: (a) a stacked column chart with relative abundances of AMR genes aggregated to corresponding ARGs (*y*‐axis) by sample (*x*‐axis) with the height of each bar chart relating to the relative AMR gene abundances in a sample and (b) heatmap showing AMR gene abundances based on the relative abundance values. The color scale represents the abundance, from red (high abundance) to blue (low abundance or no ARGs detected).

**Figure:**
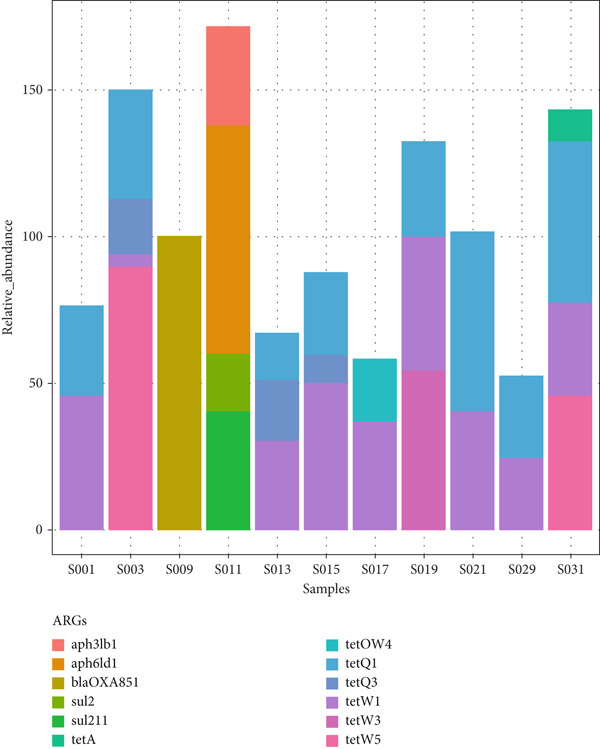
(a)

**Figure:**
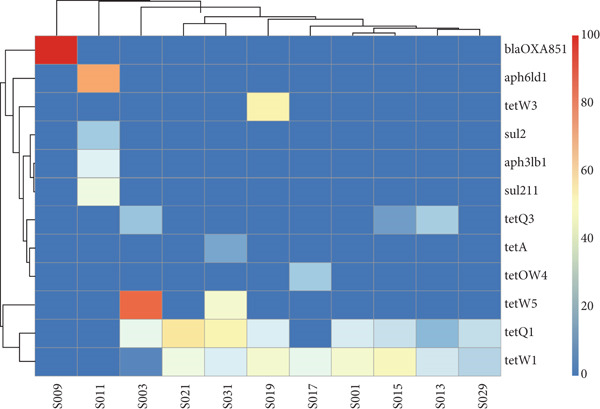
(b)

The major ARGs identified in cecal samples are shown in Figure 10, indicating that the abundance of ARGs varied among the samples. Similar to fecal samples, genes conferring resistance to tetracycline (*tetQ1*, *tetQ2*, *tetQ3*, *tetW1*, *tetW3*, and *tetW5*) were detected in higher numbers in most of the cecal samples, with *tetQ1*, *tetW1*, and *tetW5* being common across the different poultry species. Other ARGs identified included those conferring resistance to *β*‐lactamases (*bla_OXA2091_*), sulfonamides (*sul2*), and macrolides (*ermF1*, *ermF3*, and *ermG1*). These antibiotic classes were exclusively identified in chicken cecal samples. Multidrug efflux pumps (*tet44*) were also identified.

Figure 10Total level of antimicrobial resistance genes in cecal samples: (a) a stacked column chart with relative abundances of AMR genes aggregated to corresponding ARGs (*y*‐axis) by sample (*x*‐axis), with the height of each bar relating to the relative AMR gene abundances in a sample, and (b) a heatmap showing AMR gene abundances based on the relative abundance values. The color scale represents the abundance, from red (high abundance) to blue (low abundance or no ARGs detected).

**Figure:**
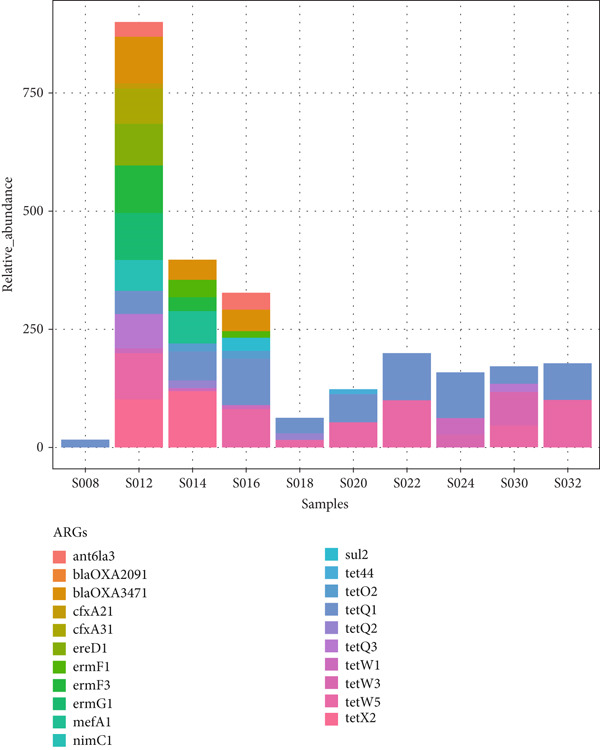
(a)

**Figure:**
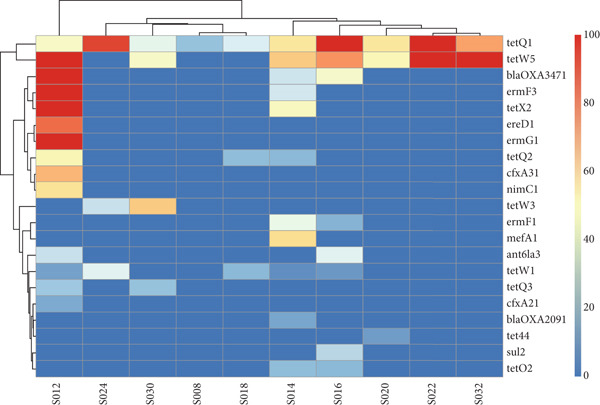
(b)

## 4. Discussion

The gut microbiota plays a crucial role in the health and productivity of poultry. Its balance directly influences growth efficiency, disease resistance, and economic outcomes [37, 38]. However, the gut microbiota profile of indigenous backyard poultry remains largely understudied. Previously, we examined the effectiveness of cloacal and oropharyngeal swabs in studying the gut microbiota of indigenous backyard poultry [22]. Here, we used fecal samples as they present a cost‐effective and noninvasive approach. This approach allows for easy sample collection and continuous monitoring of changes in the metagenome over time. Ceca were also sampled to provide a more accurate representation of the core gut microbiota. The digestion of oligosaccharides and other complex polysaccharides, along with the production of short‐chain fatty acids (SCFAs) through microbial fermentation, is thought to occur in the cecum, and these processes are closely linked with the cecal microbiota [21]. Therefore, the characterization of microbiota found in feces and ceca of indigenous backyard poultry could provide crucial information related to poultry health and production in free‐range (backyard or scavenging) production systems practiced worldwide.

The alpha diversity results showed no significant differences in bacterial species richness, diversity, and evenness within the fecal and cecal samples, as well as among the indigenous backyard poultry species. Additionally, beta analysis also showed no clear distinction between the bacterial communities in the fecal and cecal samples. A related study by Andreani [16] found no clear distinction between cloacal and cecal microbiomes in broiler chickens from Northern Ireland. This was also consistent with observations by Kang [19], who noted similarities in microbial community structures among fecal, cecal, and cloacal samples but different microbial abundances. These findings suggest that noninvasive methods, such as cloacal and fecal samples, can accurately reflect the core gut microbiota, particularly for abundant taxa. However, it is essential to exercise caution, as the microbiota in the cloaca and feces may not accurately represent that of the digestive tract due to environmental exposure [19, 39].

Our findings revealed that Bacteroidetes, Firmicutes, and Proteobacteria were the dominant phyla in both fecal and cecal samples, consistent with previous observations that reported the abundance of Firmicutes, Bacteroidetes, and Proteobacteria in poultry gut metagenomes [15, 19–21]. Our results also align with earlier studies that noted the presence of Archaea (such as Euryarchaeota) in the poultry gut, although in smaller proportions [40]. The classes of microorganisms identified in both fecal and cecal samples across the poultry species included Bacteroidia, Methanobacteria, Clostridia, Gammaproteobacteria, Epsilonproteobacteria, Bacilli, Deltaproteobacteria, Betaproteobacteria, Chlamydia, Spirochaetia, and Synergistia. These findings favorably compare with similar observations made by Bhogoju [15], who reported identical classes of bacteria in the guts of chicken and guinea fowl in the United States. We identified 37 and 33 families in fecal and cecal samples, respectively, across the poultry species, including Bacteroidaceae, Oscillospiraceae, Methanobacteriaceae, Lachnospiraceae, Clostridiaceae, Prevotellaceae, Synergistaceae, Chlamydiaceae, and Enterobacteriaceae. Bhogoju [15] reported that members of the family Lachnospiraceae were abundant in animal digestive tracts. Several members of this family contribute to the production of butyric acid, which is essential for microbial and host epithelial cell growth. Additionally, Bhogoju [15] posited that Prevotellaceae is a family of bacteria involved in breaking down proteins and carbohydrates and is commonly found in the guts of animals. Research is ongoing to explore the connection between members of the Prevotellaceae family and Parkinson′s disease. Members of the family Enterobacteriaceae are also frequently present in animal guts, primarily as commensal organisms. However, some, including *Salmonella* and *Escherichia*, are known to be pathogenic.

A total of 61 and 57 genera were identified in fecal and cecal samples, respectively, from the indigenous backyard poultry species. The dominant genera detected in cecal samples included *Bacteroides*, *Methanobrevibacter*, *Chlamydia*, *Pseudoflavonifractor*, *Elusimicrobium*, *Candidatus Alloclostridium*, *Faecalibacterium,* and *Prevotella.* In contrast, the dominant genera in fecal samples were *Bacteroides*, *Methanobrevibacter*, *Phocaeicola*, *Candidatus Adamsella*, *Mediterranea*, and *Pseudoflavonifractor*. Enterobacteria, Lactobacilli, and Enterococci were observed to dominate the chicken gut in Malaysia [9], while *Lactobacillus* and *Bacteroides* were predominant in the small intestines of chickens in China [21]. *Bacteroides* are important commensals in the poultry gut, as they play a role in the degradation of essential complex carbohydrates and produce SCFAs [41]. *Methanobrevibacter* has also been reported as the predominant archaeal genus in the poultry gut, with *Methanobrevibacter woesei* being the most prolific among the archaeal domain in poultry [40]. The roles of *Chlamydia*, *Pseudoflavonifractor*, *Elusimicrobium*, *Faecalibacterium,* and *Prevotella* as important commensal microorganisms in poultry have also been documented [15, 42–45]. It is noteworthy that comparing OTUs and taxonomic composition between the current study and other reported studies may be influenced by poultry species, production systems, and the approaches used in the study [9, 22]. However, other factors such as the environment, treatment, feed additives, antibiotics, age, horizontal gene transfer, hygiene levels, diet, poultry species, and agroclimatic considerations may also significantly impact the composition of the poultry gut microbiome [9].

The functional diversity of a microbial community can be quantified by annotating metagenomic sequences with functions. The KEGG pathway analysis revealed that functions such as metabolism and cellular processes were predicted in the fecal and cecal samples. Conversely, the COG pathway analysis indicated that cellular processes, information storage and processing, and metabolism were detected in the fecal and cecal samples. These findings align with the observations of Kumar [20] and Panyako [22], who reported that metabolism, genetic information processing, cellular processes, and organismal systems were the dominant functions predicted in the KEGG pathway analysis.

The identification and characterization of ARGs is a valuable indicator of antimicrobial use by farmers. Previously, this was achieved through the cloning of cultured bacteria, which resulted in significant losses of several potential ARGs because most bacteria are not cultivable [20]. The growing interest in AMR research is driven by concerns about the improper and unregulated use of antibiotics in many global settings, leading to the uncontrolled proliferation of ARGs, which is a major global health issue [20, 46]. Fecal samples were found to be more suitable for detecting antibiotic resistance than rectal and cecal samples [19]. In this study, several ARGs were detected in fecal samples, with the most predominant genes conferring resistance to tetracycline (*tetW3*, *tetQ1*, *tetA*, *tetW1*, and *tetW5*). However, *tetQ1* and *tetW1* were found in most poultry fecal samples, implying that these are the most common tetracycline‐resistant genes in poultry feces. Other ARGs identified in some samples included those conferring resistance to *β*‐lactamases (*bla_OXA851_*), aminoglycosides (*aph6ld1* and *aph3lb1*), and sulfonamides (*sul211*); these antibiotic classes were observed solely in chicken feces, suggesting the threat posed by AMR to indigenous backyard chickens. Similarly, the main ARGs found in cecal samples were those conferring resistance to tetracycline, such as *tetQ1*, *tetQ2*, *tetQ3*, *tetW1*, *tetW3*, and *tetW5*.

The most abundant tetracycline‐resistant genes found across various poultry species included *tetQ1*, *tetW1*, and *tetW5*. Other identified ARGs conferred resistance to *β*‐lactamases (*bla_OXA2091_*), sulfonamides (*sul2*), and macrolides (*ermF1*, *ermF3*, and *ermG1*). These additional antibiotic classes were present only in chicken cecal samples. In a similar study examining AMR in Ethiopian backyard chickens, Kumar [20] reported that the most prevalent ARGs detected were those conferring resistance to tetracycline, such as *tetQ*, *tetW*, and *tetX*. These findings also align with data from other metagenomic studies on poultry [19, 47], which underscore the threat of AMR to indigenous backyard poultry production in Kenya.

The abundance of genes conferring resistance to tetracycline may likely stem from ongoing selective pressure in the environment, given that tetracyclines constitute the most widely used class of antimicrobials in veterinary medicine and horticulture in developing nations, as previously stated by Skarżyńska et al. [47]. Skarżyńska et al. also pointed out that other ARGs commonly found in farm animals belong to antibiotic classes critical for humans, such as macrolides, aminoglycosides, and beta‐lactams. This is anticipated to result in significant antibiotic resistance among many bacterial pathogens in both humans and animals in Kenya and other developing regions. However, it is important to note that while the presence of ARGs does not automatically indicate that a particular microorganism is resistant to the corresponding antimicrobial agent, it nonetheless heightens the risk of AMR development.

A limitation of this study is that the data were obtained from pooled samples rather than from individuals. This could diminish the epidemiological strength of the study by affecting the ability to assess microbial prevalence and/or loads, as the reads might have been biased by such a procedure, as suggested by Lima et al. [12]. However, as discussed in our previous work [48], this approach offers the opportunity to access diverse microbial genomes present in hosts, including novel microorganisms. Additionally, the pooling strategy does not affect the quantitative accuracy of ARGs detected. It is also noteworthy that chickens are the primary indigenous backyard poultry species; therefore, more chicken samples were included than those of other species. This led to uneven sample sizes between chickens and other poultry species, which may have weakened the statistical power of cross‐species comparisons. Furthermore, due to resource limitations, our study did not include commercial poultry and environmental samples as controls. We recommend further research comparing the metagenomes of poultry raised in both free‐range and controlled environments (intensive and semi‐intensive) to assess how free‐range settings affect microbial communities in poultry and to determine the true source of ARGs detected, whether from within the birds themselves or from environmental exposure.

## 5. Conclusion

Several factors influence the indigenous backyard poultry gut microbiota, including host immunity, developmental stage, diet, and the history of contact with the environmental microbial community. In this study, we compared the fecal and cecal microbiota of indigenous backyard chickens, ducks, pigeons, and guinea fowl raised under free‐range conditions (either in the backyard or scavenging). Therefore, the microbial profiles reported here may have arisen from interactions between the host and microbes, the host′s diet, and exposure to the environmental microbial community. Using feces to represent the hindgut microbiota may offer limited efficacy compared to other sample types like ceca, duodenum, or gizzard. However, it still reflects the poultry gut microbiota to an extent. Here, we have established a basic reference point for the gut microbiome of indigenous backyard poultry in Kenya and other regions worldwide that practice the free‐range poultry production system.

We observed no significant difference in the microorganism diversity between fecal and cecal sample types. Additionally, we identified numerous commensal microorganisms in the poultry species studied, which could be easily utilized for probiotics, thereby providing alternatives to antibiotics in poultry production. The abundance of tetracycline resistance genes in the studied poultry raises concerns about the risks associated with the ongoing and inappropriate use of these antimicrobials in free‐range poultry production systems. The data on ARGs from this study serve as a useful indicator of antimicrobial use in indigenous backyard poultry managed by rural smallholder farmers. This information will be key in antimicrobial and diagnostic stewardship programs designed to address the rising concern of AMR linked to the use of antibiotics in food‐producing animals, particularly poultry meat. This study not only improves our understanding of the poultry gut microbiome but also provides a valuable resource for investigating antibiotic resistance in indigenous backyard poultry in Kenya and other developing countries. More importantly, it also highlights the critical significance of AMR research within the broader frameworks of global food safety, public health, and One Health.

## Conflicts of Interest

The authors declare no conflicts of interest.

## Author Contributions

Writing—original draft, formal analysis, methodology, and writing—review and editing: P.M.P., S.O., and S.N.K. Conceptualization, funding acquisition, resources, project management, methodology, and writing—review and editing: S.C.O., J.K.L., and J.M.

## Funding

The study is supported by the National Commission of Science, Technology and Innovation, National Research Fund (NRF/Newton Utafiti Fund/1/04), and the Biotechnology and Biological Sciences Research Council.

## Supporting information


**Supporting Information** Additional supporting information can be found online in the Supporting Information section. Table S1: Pairwise comparison of poultry metagenomes in different sample types using Wilcoxon rank‐sum test based on observed number of OTUs. Table S2: Pairwise comparison of poultry metagenomes in different sample types using Wilcoxon rank‐sum test based on Shannon diversity index. Table S3: Pairwise comparison of poultry metagenomes in different sample types using Wilcoxon rank‐sum test based on Chao1 diversity index.

## Data Availability

The sequencing data that support the findings of this study are publicly available in the National Center for Biotechnology Information (NCBI) database under BioProject PRJNA1020399
