# Supplementary material for: Metagenomic Profiling of Fecal and Cecal Microbiota and Their Antimicrobial Resistance Genes in Indigenous Backyard Poultry
Source: Int J Microbiol. 2026 Jan 25;2026:7306065. doi: 10.1155/ijm/7306065 (PMC12831995; doi:10.1155/ijm/7306065)
Supplement: Supplementary file 1 — Supporting Information Additional supporting information can be found online in the Supporting Information section. Table S1: Pairwise comparison of poultry metagenomes in different sample types using Wilcoxon rank‐sum test based on observed number of OTUs. Table S2: Pairwise comparison of poultry metagenomes in different sample types using Wilcoxon rank‐sum test based on Shannon diversity index. Table S3: Pairwise comparison of poultry metagenomes in different sample types using Wilcoxon rank‐sum test based on Chao1 diversity index. [file IJM-2026-7306065-s001.docx]

# Supplementary Materials: Metagenomic profiling of faecal and caecal microbiota and their antimicrobial resistance genes in indigenous backyard poultry

| **Table S1**: Pairwise comparison of poultry metagenomes in different sample types using Wilcoxon rank-sum test based on observed number of OTUs | |
| --- | --- |
|  | Caecum |
| Faeces | 0.25 |

| **Table S2**: Pairwise comparison of poultry metagenomes in different sample types using Wilcoxon rank-sum test based on Shannon diversity index | |
| --- | --- |
|  | Caecum |
| Faeces | 0.88 |

| **Table S3**: Pairwise comparison of poultry metagenomes in different sample types using Wilcoxon rank-sum test based on Chao1 diversity index | |
| --- | --- |
|  | Caecum |
| Faeces | 0.75 |
